# Relationship of Climatic and Forest Factors to Drought- and Heat-Induced Tree Mortality

**DOI:** 10.1371/journal.pone.0169770

**Published:** 2017-01-17

**Authors:** Qingyin Zhang, Ming’an Shao, Xiaoxu Jia, Xiaorong Wei

**Affiliations:** 1 College of Natural Resources and Environment, Northwest A&F University, Yangling, Shaanxi Province, China; 2 Key Laboratory of Ecosystem Network Observation and Modeling, Institute of Geographic Sciences and Natural Resources Research, Chinese Academy of Sciences, Beijing, China; 3 State Key Laboratory of Soil Erosion and Dryland Farming on the Loess Plateau, Northwest A&F University, Yangling, Shaanxi Province, China; 4 College of Resources and Environment, University of Chinese Academy of Sciences, Beijing, China; Chinese Academy of Forestry, CHINA

## Abstract

Tree mortality due to warming and drought is a critical aspect of forest ecosystem in responding to climate change. Spatial patterns of tree mortality induced by drought and its influencing factors, however, have yet to be documented at the global scale. We collected observations from 248 sites globally where trees have died due to drought and then assessed the effects of climatic and forest factors on the rate of tree mortality. The global mean annual mortality rate was 5.5%. The rate of tree mortality was significantly and negatively correlated with mean annual precipitation (*P* < 0.01). Tree mortality was lowest in tropical rainforests with mean annual precipitation >2000 mm and was severe in regions with mean annual precipitation <1000 mm. Mortality rates varied amongst species. The global annual rate of mortality was much higher for gymnosperms (7.1%) than angiosperms (4.8%) but did not differ significantly between evergreen (6.2%) and deciduous (6.1%) species. Stand age and wood density affected the mortality rate. Saplings (4.6%) had a higher mortality rate than mature trees (3.2%), and mortality rates significantly decreased with increasing wood density for all species (*P* < 0.01). We therefore concluded that the tree mortality around the globe varied with climatic and forest factors. The differences between tree species, wood density, stand density, and stand age should be considered when evaluating tree mortality at a large spatial scale during future climatic extremes.

## Introduction

Warm droughts, one of the most important global climate changes, have recently occurred in North America, Africa, Europe, Amazonia, and Australia, with major effects on terrestrial ecosystems, carbon balances, and food security [1, 2]. Warm droughts can alter ecosystemic structure, composition, and services, such as carbon sequestration, biological conservation, and water regulation [3–6]. Prolonged droughts or heat can kill trees [7]. Recent studies have indicated that forest mortality induced by rising temperatures and increased drought have rapidly increased around the world during the past decade [6–9]. For example, severe drought has led to massive mortality of aspen along the southern edge of the Canadian boreal forest [10]. van Mantgem et al. [3] showed that increases in the rates of tree mortality in the western United States could not be attributed solely to endogenous increases in competition or to the aging of large trees. Regional warming and the consequent increases in water deficits are likely contributors to the increases in mortality rates. Pervasive tree growth has declined in the semi-arid forests of central Asia since 1994 due to warming-induced increases in the demand for atmospheric moisture [11]. Continued warming in central Asia would likely further increase forest stress and tree mortality, potentially driving the eventual loss of regional semi-arid forests. Drought stress can also decrease the self-organisation of forest ecosystems and consequently increase vulnerability to climate change [12]. Drought- and heat-induced tree mortality have occurred globally, and the effects of extreme climatic events, such as heat waves, drought, and flooding, on plant growth and mortality have been reviewed [6,7], but the spatial patterns of tree mortality induced by drought and heat around the world have yet to be determined.

Drought-induced tree mortality can be influenced by various factors, such as climatic and biotic factors [13–15]. Campos et al. [1] showed that the patterns of response to drought are strongly associated with annual precipitation across biomes, indicating an intrinsic systemic sensitivity to water availability across annual precipitation regimes. Clifford et al. [13] found thresholds of annual precipitation and vapour-pressure deficit (VPD) for tree mortality in southwestern North America. They showed that the patterns of *Pinus edulis* die-off had threshold responses to annual precipitation and VPD, with little to no mortality (<10%) above 600 mm and below a warm-season VPD of *ca*. 1.7 kPa. Tree mortality always accompanies warmer temperatures triggered by increasingly severe drought. Adams et al. [14] showed that an increase in temperature of 4.3°C shortened the time to drought-induced mortality in *P*. *edulis* and that this effect of temperature also predicted a 5-fold increase in the frequency of tree deaths in the southwestern United States. Therefore, we hypothesized that tree mortality decreased with increasing annual precipitation but increased with increasing annual temperature.

Regional-scale die-off of trees may also be controlled by biotic factors such as tree species, wood density, stand density, and size class [15–19]. Demographic changes, such as decreasing stand density, would greatly favour reductions in the rate of tree mortality compared to denser stands [16]. Larger trees were confronted with a stronger mortality than smaller trees in tropical rainforests [15], contrasting with the drought-related mortality in North America, where smaller trees encountered more risk than larger trees [3, 20]. Kraft et al. [18] found that mortality rates declined with increasing wood density at half of their study sites, but the relationship varied amongst families, plots, and even census intervals within sites. These biotic factors may therefore affect the response of tree mortality to drought at a large spatial scale, which needs further examination. Thus, we hypothesized that response of tree mortality to warming and drought shifted with these biotic factors.

The objective of this study was to investigate the relationships of mean annual precipitation (MAP), mean annual temperature (MAT), elevation, the standardized precipitation-evapotranspiration index (SPEI), elevation, species, tree density, wood density and stand age to heat- drought induced mortality. We collected observations from 62 peer-reviewed publications to create a global data set of 248 sites where tree deaths have been reported.

## Materials and Methods

### Data preparation

We performed a three-pronged search of the literature on drought and tree mortality. First, we searched Google Scholar and Web of Science using the keywords drought, forest, tree, vegetation, mortality, and dieback. The literature conformed to our study published up to 2016. We then drew on references cited in several extensive peer-reviewed syntheses that comprehensively documented studies reporting drought-induced tree mortality at a regional scale [7, 8, 15]. Finally, we paid close attention to the IPCC 2014 Working Group II Bureau report that discussed the globally increasing forest mortality due to drought and heat stress but without specifying rates of tree mortality [21]. All data for mortality rates were either obtained from tables or extracted by digitising graphs using Get Data Graph Digitizer (ver. 2.25.0.32, Russia).

To be included in the meta-analysis, the study had to follow the criteria below:

1. specific tree mortality rates, with drought attributed as a prominent or the dominant driver;
2. indicate that no other disturbance such as fire or harvesting had occurred that could induce tree mortality;
3. drought episodes must be associated with natural droughts rather than drought experiments.

Attributing mortality to drought is not always straightforward, so we included only studies that stated or demonstrated that drought had driven elevated mortality rates for at least some species or region in the study. We restricted this meta-analysis to droughts shorter than five years [15]. We were limited to 62 of 64 studies that focused on specific short-term (≤5 years) droughts, suggesting a higher confidence that drought was the dominant signal in these studies. Sheil and May [22] provided a mathematical proof that estimates of mortality rates for heterogeneous populations decreased as census interval increased, so we removed the two studies that reported specific mortality rates for long-term droughts. The remaining 62 studies included 248 sites in 30 countries around the world for 1985–2016 and hundreds of species across a range of biomes, including tropical rainforests, temperate deciduous and evergreen forests, boreal forests, and savannah woodlands (S1 File).

### Calculation of mortality rate

Tree mortality was generally reported in two ways, depending on the publication. The first was the annual change in mortality (% y^-1^). For a population experiencing mortality as a constant fraction *m*_*y*_ (0 ≤ *m* ≤ 1) each year, the mortality rate after *t* years compounds as [22]:

$$m_{y}=1-{\left(N_{t}/N_{0}\right)}^{1/t}$$ (1)

where *m*_*y*_ is an approximation of the annual mortality rate (% y^-1^), and *N*_*0*_ and *N*_*t*_ are population counts at the beginning and end of a census interval or drought *t*, respectively.

The second was the total mortality rate during droughts (%). For a population experiencing mortality at a constant fraction *m* (0 ≤ *m* ≤ 1), the mortality rate after *t* years compounds as [22]:

$$m=(N_{0}-N_{t})/N_{0}$$ (2)

where *m* is an approximation of the total mortality rate during droughts (%), and *N*_*0*_ and *N*_*t*_ are as above.

Estimates of mortality rate are potentially sensitive to the census interval or drought over which they are calculated because of the non-standard turnover rates. To allow comparison of the tree mortality data, we transformed the data between total mortality rate during drought (%) and annual mortality rate (% y^-1^) based on the tree mortality of droughts as [22]:

$$m_{y}=1-{\left(1-m\right)}^{1/t}$$ (3)

$$m=1-{\left(1-m_{y}\right)}^{t}$$ (4)

### Mortality analyses

We collected a variety of data for each site, which are important for estimating mortality at a regional scale [3, 13, 23]. Specifically, we collected data for mean annual precipitation (MAP), mean annual temperature (MAT), the standardized precipitation-evapotranspiration index (SPEI), elevation, angiosperm or gymnosperm, evergreen or deciduous leaf habit, wood density, stand age, and stand density. The MAP and MAT data were obtained from the publications and http://www.worldweather.cn/zh/home.html, and the SPEI data were obtained from SPEIbase (http://sac.csic.es/spei/database.html), at a spatial resolution of 0.5° for each site. We further determined the relationship between tree mortality rate and SPEI in the drought periods based on the meteorological data in each study. The effects of droughts can be quantified using the SPEI at different time scales [24]. The SPEI is derived using an original Standardized Precipitation Index calculation procedure (SPI) [25], based on a precipitation probabilistic approach. This parameter represents a simple climatic water balance obtained at different time scales. In this study, the values of SPEI during drought periods was used by calculating at monthly scale.

The hundreds of tree species in the data set were generally divided into two groups, angiosperms and gymnosperms, for comparisons of hydraulic traits and mechanisms for mortality prediction [23]. The differences in phenological leaf habit play an important role in the response of plant growth to drought [26, 27], so we also assessed the relationship between mortality rate and phenological leaf habit (evergreen and deciduous).

Calculations of mortality rates are often limited by stand age. For example, some studies rated stand age as adults and saplings, using diameter at breast height (DBH), or directly provided the age of stand establishment. We examined the effects of stand age on drought-induced tree mortality by dividing the species with specific mortality rates into two main sizes: saplings (reported in the publications as saplings, seedings, young stands, or DBHs <20 cm) and adults (reported as adults, old stands, or DBHs ≥20 cm). Twenty of the studies reported correlations between mortality rate and stand age.

We also analysed the relationship between stand density and mortality rate despite limited data for 10 of the publications. Wood density was defined as the dry weight per unit volume of wet wood. The data for wood density was obtained from the Global Wood Density Database [28]. Two studies reported mortality rates only at the genus level, so we calculated generic averages for hundreds of species from the Global Wood Density Database. Eight studies that reported average mortality rates for hundreds of species have no data of specific wood density. Using global values for traits for a given species at specific sites or regions presents challenges, so we used the study-specific values for species, when reported, to improve accuracy [23]. The agreement between our findings for the average role of wood density in predicting cross-species mortality patterns and those of detailed studies across multiple biomes that directly measured tree traits indicated that our approach was reasonable [29, 30].

We also divided the data into nine climatic types to compare the patterns of tree mortality across different climatic regions: tropical rainforest, tropical monsoon, tropical savannah, subtropical monsoon, Mediterranean, temperate maritime, temperate continental, temperate monsoon, and alpine. Only climatic types with >10 sites with specific mortality rates were analysed.

The effect of drought on biomass followed an approximately linear relationship with drought intensity [15], however, we had no priori reason to expect such a linear relationship to be universally true, and the current data sets may include sites that subjected to drought more extensively. Mortality rate could increase rapidly once a certain drought stress threshold is passed [13], inconsistent with our expectation that forests might be resilient to modest drought. We therefore adopted a curve-fitting procedure, examining best fits for linear, log, exponential, and three-factor polynomial relationships.

### Statistical analysis

The various analytical combinations required that we develop multiple models. We initially considered 48 statistical models based on 3 groups (angiosperms, gymnosperms, and all data) × 2 mortality metrics (annual mortality rate (% y^-1^) and total mortality rate during droughts (%)) × 2 drought metrics (MAP and SPEI) × 4 linear and various nonlinear relationships). We then selected the best models for each of the group⁄mortality⁄drought combinations on the basis of *R*^2^ and Akaike’s information criterion (AIC) [15] and calculated 95% confidence intervals for the lines of best fit. For polynomial models, we fitted all possible two- and three-factor models and only selected a model with cubic terms when it had an AIC lower than all other models (except the exponential model).

All values in this study represent the mean ± SE. Multi-way analyses of variance (ANOVAs) tested the effects of the differences amongst MAP, MAT, and elevation and between forest type and wood density. Stepwise regression analysis was used to assess the relationship between tree mortality rate and elevation, MAP, MAT, SPEI, and wood density. All statistical analyses were performed using SPSS 18.0 (SPSS for windows, Chicago, IL, USA).

## Results

### Tree mortality rates around the globe

**Fig 1:**
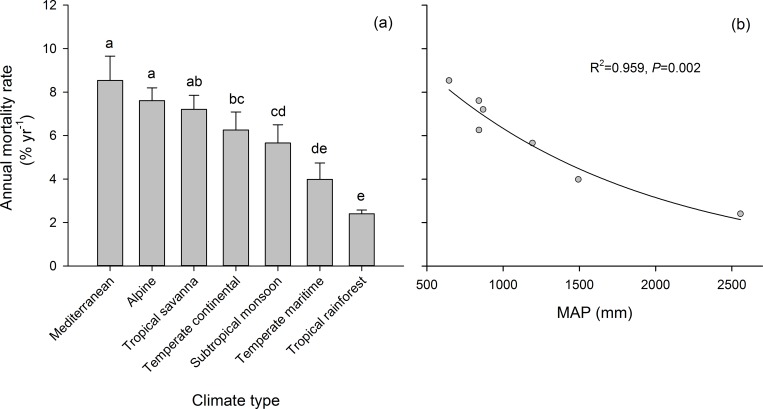
(a) Annual mortality rates for the climatic types. Significant differences between the annual mortality rates of the climatic types are labelled with different lowercase letters (*P* < 0.05). Values represent the mean ± SE. (b) Relationship between annual mortality rate and mean annual precipitation (MAP).

The average annual rate of drought-induced tree mortality was 5.5 ± 0.3% from 248 sites around the world. Only seven of the nine climatic types met our requirement of >10 sites. The annual mortality rate (8.5 ± 1.1%) was highest in the Mediterranean zones of southern Europe, followed by alpine (7.6 ± 0.6%), tropical savannah (7.2 ± 0.6%), temperate continental (6.3 ± 0.8%), subtropical monsoon (5.7 ± 0.8%), and temperate maritime (4.0 ± 0.8%) zones, and the rate was lowest in the tropical rainforest zones of the Amazon Basin (2.4 ± 0.2%) (Fig 1A). The annual mortality rate exponentially decreased with annual precipitation across the climatic zones (*P* < 0.001, Fig 1B).

### Effects of biotic and climatic factors

The annual mortality rate was significantly higher for gymnosperms (7.1 ± 0.5%) than angiosperms (4.8 ± 0.4%) (Table 1). Mortality rate, however, did not differ significantly between evergreen and deciduous forests. The rate was higher for saplings (4.6 ± 0.5%) than adults (3.2 ± 0.6%), but not significantly.

**Table 1:** Mean tree mortality rates and wood densities by tree group, forest type, and stand age. Significant differences between biotic factors (angiosperm or gymnosperm, deciduous or evergreen, sapling or adult) are labelled with different lowercase letters (*P <* 0.05).

Biotic factor	Number of plots	Wood density (g cm^-3^)	Annual mortalityrate (% y^-1^)	Mortality rate during droughts (%)
		Mean ± SE	Mean ± SE	Mean ± SE
**Angiosperm**	106	0.562 ± 0.14a	4.8 ± 0.4b	13.9 ± 1.1b
**Gymnosperm**	104	0.396 ± 0.07b	7.1 ± 0.5a	21.4 ± 1.1a
**Deciduous**	40	0.492 ± 0.11a	6.1 ± 0.7a	17.4 ± 1.9a
**Evergreen**	160	0.466 ± 0.14a	6.2 ± 0.4a	18.8 ± 1.0a
**Sapling**	34	0.449 ± 0.11a	4.6 ± 0.5a	18.2 ± 1.9a
**Adult**	45	0.459 ± 0.10a	3.2 ± 0.6b	13.5 ± 1.9b
**All**	248	0.479 ± 0.14	5.5 ± 0.3	15.9 ± 0.8

**Fig 2:**
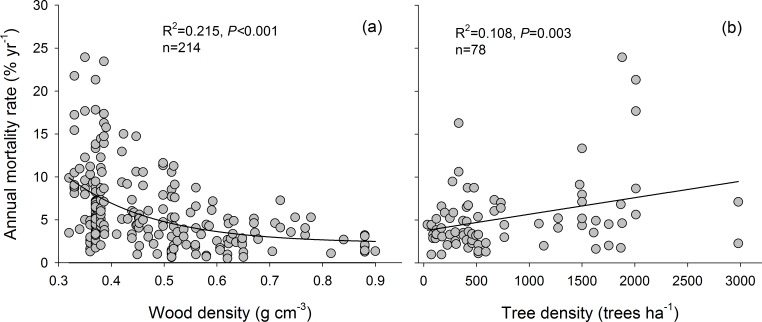
Relationships between annual mortality rate and (a) wood density and (b) tree density.

The annual mortality rate for all species decreased significantly with increasing wood density (Fig 2A) and increased significantly with stand density (Fig 2B). Group and forest type both had significant interactive effects with wood density, but the interactive effects between group and forest type and between group, forest type, and wood density were not significant (Table 2).

**Table 2:** Multi-way analysis of variance of tree mortality rate for mean annual precipitation (MAP), mean annual temperature (MAT), elevation, group, forest type, and wood density. Group includes angiosperm and gymnosperm species; forest type includes evergreen and deciduous species.

Parameter	*df*	*F*	*P*
**MAP**	4	8.936	0.000
**MAT**	3	0.440	0.724
**Elevation**	4	2.132	0.078
**MAP × MAT**	6	1.866	0.088
**MAP × Elevation**	8	1.828	0.073
**MAT × Elevation**	7	6.596	0.000
**MAP × MAT × Elevation**	7	3.058	0.004
**Group**	1	2.048	0.045
**Forest type**	1	0.074	0.650
**Wood density**	3	5.166	0.002
**Group × Forest type**	1	0.560	0.349
**Group × Wood density**	3	3.889	0.010
**Forest type × Wood density**	3	4.860	0.003
**Group × Forest type × Wood density**	1	2.163	0.143

**Fig 3:**
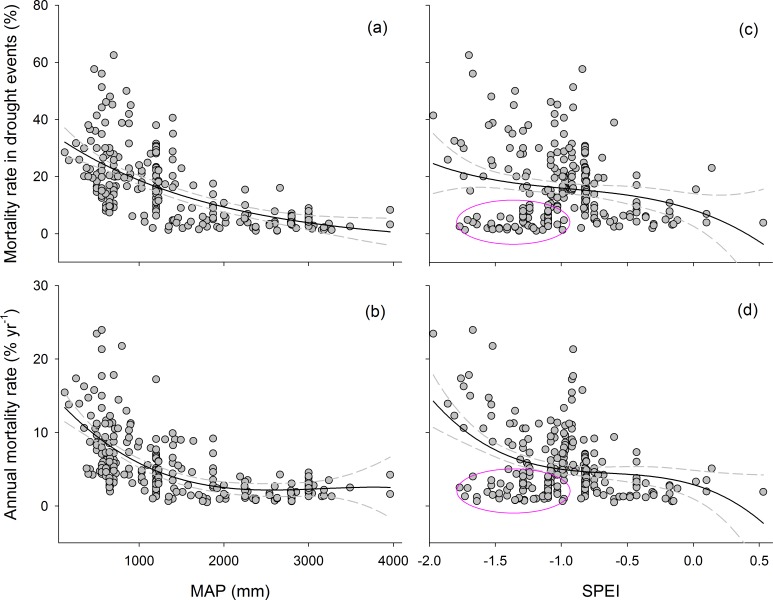
Sensitivity of mortality to drought for all sites with available data. (a) and (b) showed relationships between tree mortality metrics and MAP. (c) and (d) showed relationships between tree mortality metrics and SPEI. Dark-grey dots indicate droughts. The best-fit models for each drought index and mortality-rate metric are shown, with the 95% bootstrapped confidence intervals. MAP, mean annual precipitation; SPEI, standardized precipitation-evapotranspiration index. The pink ovals indicate the lower tree mortality rate for tropical rainforests.

The tree mortality rate was significantly and negatively correlated with MAP around the world (*P* < 0.01) (Fig 3A and 3B). The mortality rate was higher (8.6 ± 0.5%) in regions with MAPs <1000 mm and lower (2.1 ± 0.1%) in tropical rainforests with MAPs >2000 mm.

Annual mortality rate was not significantly correlated with MAT (*P* = 0.724) or elevation (*P* = 0.078) (Table 2) but was significantly affected by their interaction (*P* < 0.01). The effect of changes in SPEI on the rate varied amongst the regions. For example, the rate was low in Amazonia where SPEI was low (pink ovals in Fig 3C and 3D).

### Variation of tree mortality rate with multiple factors

The multifactorial statistical model explained ~47% of the variance in annual mortality rates for all data sets (Table 3). Some of the unexplained variation may have been caused by our inability to include topoedaphic and plant community effects or those of other potentially useful traits because of the lack of data. The main factors that contribute to heat and drought induced mortality varied with tree group or forest type. Elevation, MAP, SPEI, and wood density were the main factors for angiosperms and evergreen forests, but elevation was the only main factor for deciduous forests. Elevation, MAP, SPEI, MAT, and wood density has the most effect on gymnosperm mortality. Elevation, MAP, SPEI, and wood density were the main factors for all species combined.

**Table 3:** Stepwise regression to identify factors (elevation, mean annual precipitation, mean annual temperature, standardized precipitation-evapotranspiration index, and wood density) determining the annual mortality rate during droughts. M, annual mortality rate during droughts; E, elevation; P, mean annual precipitation; T, mean annual temperature; S, standardized precipitation-evapotranspiration index; W, wood density.

Tree group/type	Equation	*R*^2^	*P*	n
**Angiosperm**	M = 0.002E-0.001P-2.455S-6.233W+7.5	0.608	0.000	106
**Gymnosperm**	M = -0.002E-0.003P-6.994S-0.109T-28.097W+18.4	0.592	0.000	104
**Evergreen**	M = -0.001E-0.003P-4.436S-10.052W+11.3	0.508	0.000	160
**Deciduous**	M = 0.003E+3.6	0.452	0.000	40
**All**	M = -0.001E-0.002P-4.773S-8.575W+9.1	0.471	0.000	214

**Fig 4:**
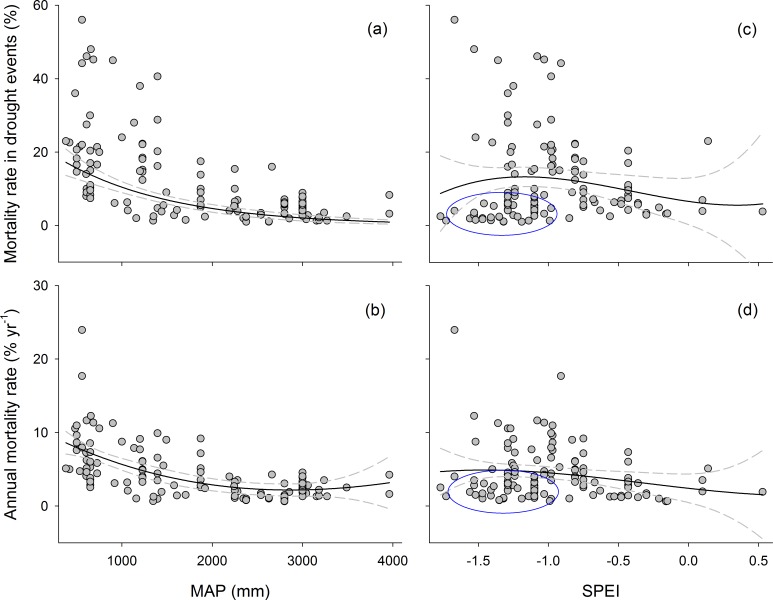
Sensitivity of mortality to drought for angiosperms. (a) and (b) showed relationships between tree mortality metrics and MAP. (c) and (d) showed relationships between tree mortality metrics and SPEI. Dark-grey dots indicate droughts. Dark-grey dots indicate droughts. The best-fit models for each drought index and mortality-rate metric are shown, with the 95% bootstrapped confidence intervals. MAP, mean annual precipitation; SPEI, standardized precipitation-evapotranspiration index. The blue ovals indicate the lower tree mortality rate for tropical rainforests.

**Fig 5:**
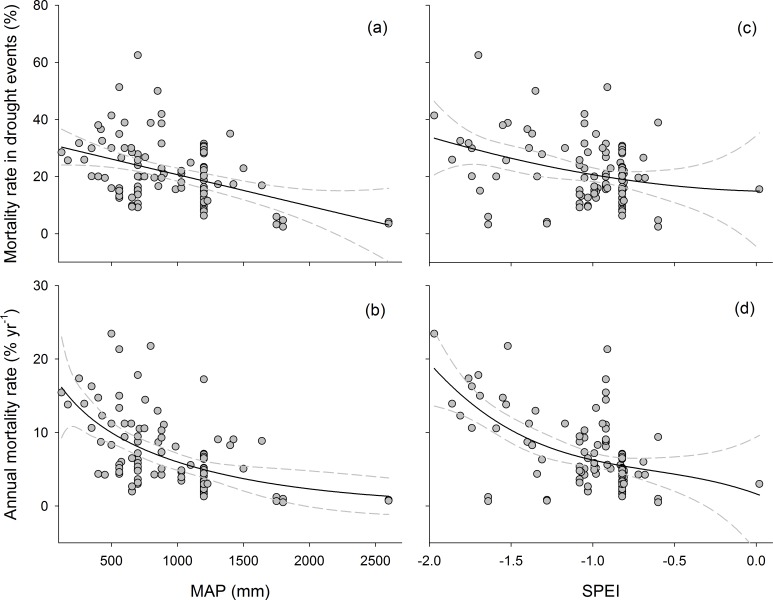
Sensitivity of mortality to drought for gymnosperms. (a) and (b) showed relationships between tree mortality metrics and MAP. (c) and (d) showed relationships between tree mortality metrics and SPEI. Dark-grey dots indicate droughts. Dark-grey dots indicate droughts. The best-fit models for each drought index and mortality-rate metric are shown, with the 95% bootstrapped confidence intervals. MAP, mean annual precipitation; SPEI, standardized precipitation-evapotranspiration index.

As expected, mortality rate decreased with increasing MAP and SPEI (Table 4 and Figs 3–5). The relationship for the entire data set was nonlinear: the rates tended to decrease disproportionately. These results indicated biome-wide sensitivity to drought, but mortality was lowest for Amazonia and Borneo (pink and blue ovals in Figs 3C and 3D and 4C and 4D). Nonlinearity produced better fits than linearity–AICs are optimal for models with a cubic term. The tropical forests in Amazonia and Borneo were less drought-sensitive than other forests, which was suggested by the displacement of the best fit across the range of drought (Fig 3C and 3D). MAP resulted in a slightly improved fit compared with SPEI for most models (Table 4). *R*^2^ tended to be higher, and lower AIC tended to be lower, for comparable models for all data sets.

**Table 4:** Model fits for the global response of tree mortality to drought. Data sets varied with tree group (angiosperm and gymnosperm), tree mortality metric (annual mortality rate (% y^-1^) and mortality rate during droughts (%)), and drought metric (MAP and SPEI). Best-fit models are highlighted in bold and are displayed graphically in Figs 3, 4 and 5. For polynomial models, we fitted all possible two- and three-factor models and only selected a model with cubic terms when it had an AIC lower than all other models and an *R*^2^ higher than all other models. MAP, mean annual precipitation; SPEI, standardized precipitation-evapotranspiration index; AIC, Akaike’s information criterion.

All data (MAP)	*R*^2^	AIC	Angiosperms (MAP)	*R*^2^	AIC	Gymnosperms (MAP)	*R*^2^	AIC
Mortality rate during droughts (%)			Mortality rate during droughts (%)			Mortality rate during droughts (%)		
Linear	0.385	2498	Linear	0.362	1191	Linear	0.197	960
Log	0.389	2496	Log	0.385	1186	Log	0.153	968
**Exponential** (Fig 3A)	**0.514**	**1156**	**Exponential** (Fig 4A)	**0.432**	**540**	**Exponential** (Fig 5A)	**0.328**	**339**
Polynomial	0.410	2487	Polynomial	0.392	1185	Polynomial	0.192	963
Annual mortality rate (% y^-1^)			Annual mortality rate (% y^-1^)			Annual mortality rate (% y^-1^)		
Linear	0.314	2018	Linear	0.321	899	Linear	0.279	787
Log	0.392	1988	Log	0.362	891	Log	0.312	782
Exponential	0.397	1150	Exponential	0.352	502	**Exponential** (Fig 5B)	**0.427**	**382**
**Polynomial** (Fig 3B)	**0.401**	**1984**	**Polynomial** (Fig 4B)	**0.372**	**889**	Polynomial	0.315	779
All data (SPEI)	*R*^2^	AIC	Angiosperms (SPEI)	*R*^2^	AIC	Gymnosperms (SPEI)	*R*^2^	AIC
Mortality rate during droughts (%)			Mortality rate during droughts (%)			Mortality rate during droughts (%)		
Linear	0.044	2607	Linear	0.013	1247	Linear	0.107	974
Log	—	—	Log	—	—	Log	—	—
Exponential	0.006	1332	Exponential	0.003	612	Exponential	0.050	374
**Polynomial** (Fig 3C)	**0.048**	**2606**	**Polynomial** (Fig 4C)	**0.025**	**1246**	**Polynomial** (Fig 5C)	**0.111**	**973**
Annual mortality rate (% y^-1^)			Annual mortality rate (% y^-1^)			Annual mortality rate (% y^-1^)		
Linear	0.116	2080	Linear	0.034	944	Linear	0.297	785
Log	—	—	Log	—	—	Log	—	—
Exponential	0.065	1259	Exponential	0.026	557	Exponential	0.153	424
**Polynomial** (Fig 3D)	**0.147**	**2071**	**Polynomial** (Fig 4D)	**0.038**	**944**	**Polynomial** (Fig 5D)	**0.322**	781

## Discussion

Drought-induced tree mortality varied globally. The spatial variation in tree mortality can be ascribed to the variations in climate and properties of the vegetation [3, 4]. The mortality rate was significantly and negatively correlated with MAP globally (Fig 3). Mortality was generally severe in regions with MAPs <1000 mm and lower in regions with MAPs >2000 mm. A higher MAP in tropical rainforests can buffer the negative effects of short-term severe drought, decreasing mortality rates in these regions [15]. SPEI was also low but with lower rates in tropical rainforests (the pink ovals in Fig 3C and 3D), suggesting that trees in these forests that are normally wettest may be less vulnerable to higher dry-season deficits than normal [31]. Conversely, the drier the long-term climate, the greater the impact of a given increase on tree mortality rate. The high mortality rates in the regions with MAPs <1000 mm may also be ascribed to uneven seasonal distributions of rainfall, highlighted by the occasional variation in the spatial extent of tree mortality (29%) during the drought in the late 1990s in northeastern Australia [32].

Mortality rate was negatively correlated with drought regardless of the mortality and drought metrics used (Table 4 and Figs 3–5). A three-factor polynomial or exponential relationship clearly provided the best fits in all four modelled fits for the entire data set, suggesting nonlinear responses of forests to drought and a possible threshold zone beyond which mortality is high, but the current data set is not yet sufficiently representative across all regions to state this proposal with confidence. Clifford et al. [13], however, reported that the patterns of pinyon die-off indicated threshold responses to annual precipitation, with little to no tree mortality (<10%), above 600 mm, in southwestern North America. A threshold for survival associated with annual precipitation could be explained by the survival of many tree species at the limit of tolerable potential water deficits [2].

Mortality rate was not significantly correlated with elevation at the global scale, consistent with the findings by Ganey and Vojta [33]. The rate, however, increased with elevation at a regional scale [3, 4]. For example, the loss of tree cover across an elevation gradient of a pinyon-juniper woodland in southwestern United States was greater at higher elevations [34]. Water stress created by regional droughts may be a dominant contributor to widespread increases in mortality rates, which increased with elevation [4].

Mortality rate was not significantly correlated with MAT, which is inconsistent with the findings for tropical forests in the Amazon Basin [15, 35] and for temperate forests in the western United States [3]. We also did not find a temperature threshold for tree mortality at the global scale. Adams et al. [14], however, reported that experimentally induced warmer temperatures (≈4°C) shortened the time to drought-induced mortality in *P*. *edulis* trees by nearly a third. Also, a 5-fold increase in the frequency of events inducing *P*. *edulis* mortality accompanied a 4.3°C increase in temperature in a 103-year record of regional drought duration [14]. The different scopes of the study areas and high temperature sensitivity in drought-induced mortality may have contributed to this discrepancy [36]. Temperature patterns within a region can also influence tree die-off, contributing to its uncertainty when other factors, such as stand demography and landscape heterogeneity, come into play [13].

Stand density, a predictor of drought mortality risk [33], has important implications for forest management [34]. Powers et al. [19] showed that thinning significantly influenced mortality as a function of density. Stand density in our study had a significantly positive effect on mortality rate globally, as expected. Forests begin to self-thin to maintain tree densities around 60% for survival [19]. Greenwood and Weisberg [37] found that mortality rate across multiple spatial scales was higher at sites with higher stand densities. Santos and Whitham [38] also showed by predictive modelling that high density would lead to higher tree mortality. The effects of annual precipitation on tree mortality may thus be regulated by stand density; forests with lower densities may be more resistant to drought stress. Water stress may explain the effect of self-thinning, but the mechanisms underlying density-dependent mortality may be uncertain due to the direct intraspecific competition. Tree mortality is expected to be high in dense stands, because high stand density typically increases moisture stress and competition amongst trees [39] and predisposes trees to attack by bark beetles [40]. Conifer mortality, however, increased slightly with decreasing density or basal area in southwestern United States during climatic stress, across both space [41] and time [3, 42]. The latter finding is potentially important, because it may indicate that thinning high-density stands would not significantly reduce tree mortality during severe drought [33]. The level of drought-induced mortality due to the climatic drivers in these studies was great enough to mask the effects of density-dependence that might have been apparent at lower levels of mortality [3, 43]. The results of our and other studies have thus highlighted the complexities of density-dependent mortality resulting in temporal and spatial variation amongst stands [33].

Low wood density was a significant predictor of the risk of drought mortality in angiosperms but not gymnosperms, perhaps because of their fundamentally different wood anatomies, with gymnosperms having more negative P50 values (the water potentials at which 50% of hydraulic conductivity is lost because of embolism), more conservative stomatal responses, and larger hydraulic safety margins (between the typical minimum xylem water potential and 50%) [2, 43]. Wood density may also be a valuable proxy for mortality risk amongst angiosperms in particular, as has been observed previously by a meta-analysis across 475 species [23] and in tropical biomes [18]. Measures of wood density were lacking in our study, so we used species-level means to estimate wood density, which may have affected the relationship with vulnerability to moisture deficits, where individual-level wood structural properties may diverge significantly from species means [44]. The functions in angiosperms of resprouting from branch nodes below dead segmented tissues and the higher amount of parenchyma in stem wood linked to their higher storage capacity (for water and non-structural carbohydrates) likely increase the ability to recover from drought [23]. Parenchyma, however, would provide little tolerance to stresses in gymnosperms, likely because of correlations with other traits not identified here, such as low specific hydraulic conductivity or the ability to regrow xylem after embolism [45]. Gymnosperms also tend to have less parenchyma or non-structural carbohydrates in their wood than angiosperms [43]. The ability to rapidly repair embolisms may rely on the presence of nearby parenchymal cells [46], which could explain the need for greater safety margins in gymnosperm than angiosperm wood [43]. In addition, annual mortality rates did not differ significantly across all studies between evergreen (6.2%) and deciduous (6.1%) species (Table 1), demonstrated for example by a meta-analysis of mortality rates across 475 species [23]. The variation of non-structural carbohydrates may be extremely similar between deciduous and evergreen species and not differ significantly seasonally [27].

Stand age tightly correlated with growth and reproduction under normal conditions with no severe drought stress or biotic agents [47]. Mortality rates in our study differed significantly between saplings and adults (Table 1). However, the annual mortality rate of both saplings and mature trees is lower than the global mean rate of 5.5%. This lower rate might be ascribed to the low amount sites of saplings and mature trees, which cannot be compared with the global mean mortality rates. Saplings responded differently from adults to extreme drought in the field and sometimes suffered higher mortality rates [48, 49]. Vulnerability to drought-related mortality and associated carbon and water fluxes varies with stand age due to the physiological and environmental (e.g. microclimate and nutrients) differences [50]. Mortality rate was higher for saplings than adults for the combination of elevation and stand age [51]. The greater sensitivity of saplings is presumably a factor driving the biomass-drought relationship [3]. Saplings or small trees often have limited root networks and so are more vulnerable to acute water stress than mature or large trees with deeper or more extensive root networks [52]. Small trees or saplings also have much smaller reserves of stored non-structural carbohydrates [53], rendering them also potentially more vulnerable to carbon stress. In addition, foliar dieback is generally controlled by hydraulic factors in response to drought [54]. For example, potted coniferous saplings experienced extreme hydraulic damage [48], but adult juniper trees during either natural [55] or experimental [56] drought experienced little hydraulic damage. Floyd et al. [57] reported that larger or older trees were often more prone to drought-induced mortality, inconsistent with our results, but this relationship was species-dependent. Phillips et al. [15] also showed that large size conferred a strong penalty in Amazonia under drought conditions but also in Borneo where drought was more severe. The mechanism may set an ultimate limit on the stand-level biomass of tropical rainforests [58]. Adults should also be at risk, especially from stronger droughts, consistent with theoretical predictions that invoke hydraulic limitations as the dominant limiter of tree height [59]. The lower gas exchange in taller trees has been proposed to explain age-related declines in tree growth [60].

Much effort is currently focused on resolving the mechanistic details associated with mortality, including the interrelationships between hydraulic failure, carbon starvation, and biotic agents [2, 9, 61]. Furthermore, studies of annual precipitation, soil moisture, and plant water potential and conductance associated with plant hydraulics and carbon metabolism are emerging [14, 56, 62]. Annual precipitation and related metrics such as SPEI or Palmer Drought Severity Index (PDSI), in association with forest data, are nonetheless the most widely accessible data for identifying the key aspects of the thresholds of drought mortality [63]. Our meta-analysis indicates that the study of multiple factors is a promising avenue for modelling mortality but also highlights some of the gaps in our understanding. Firstly, estimates of mortality rate are potentially sensitive to the census interval because of the different turnover rates of subpopulations. Mortality rates should be adjusted to a standard interval of one year by applying a generic census-interval adjustment [64]. Secondly, many experimental droughts have been attempted in some regions. However, the results from these studies suggest that the experimental forests are susceptible to drought [15]. Thus, tree mortality from experimental droughts should not be compared with the episodes associated with natural droughts. Finally, comparisons amongst sites and regions or trees and species are also complicated by biogeographic and biotic factors that can produce inconclusive results.

## Supporting Information

S1 File Drought episodes and corresponding mortality rates for all the data. (XLSX)
